# Remote Postoperative Epidural Hematoma after Subdural Hygroma Drainage

**DOI:** 10.1155/2010/417895

**Published:** 2010-07-07

**Authors:** Wellingson Silva Paiva, Arthur Maynart Pereira Oliveira, Almir Ferreira de Andrade, Roger Schmidt Brock, Manoel Jacobsen Teixeira

**Affiliations:** Division of Neurosurgery, University of São Paulo, 01416001, Brazil

## Abstract

*Objective*. Subdural hygroma is reported to occur in 5%–20% of all patients with closed head trauma, the treatment is controversial and in symptomatic cases surgical drainage is need. We report on a new case with remote acute epidural hematoma (AEH) after subdural hygroma drainage. *Case Presentation*. A 38-year-old man suffered blunt head trauma and had diffuse axonal injury grade III in CT scan. A CT scan that was late performed showed an increasing subdural fluid collection with mild mass effect and some effacement of the left lateral ventricle. We perform a trepanation with drainage of a hypertensive subdural collection with citrine aspect. Postoperative tomography demonstrated a large left AEH. Craniotomy and evacuation of the hematoma were performed. *Conclusion*. The mechanism of remote postoperative AEH formation is unclear. Complete reliance on neurologic monitoring, trust in an early CT scan, and a relative complacency after an apparently successful initial surgery for hygroma drainage may delay the diagnosis of this postoperative AEH.

## 1. Introduction

Subdural hygroma (SDG), a common complication of blunt head trauma, is reported to occur in 5%–20% of all patients with closed head trauma, the treatment is a controversial and in symptomatic cases surgical drainage is need [1, 2]. No case of acute epidural hematoma (AEH) after hygroma drainage has reported. We report on a new case with this complication after subdural hygroma drainage.

## 2. Case Presentation

**Figure 1:**
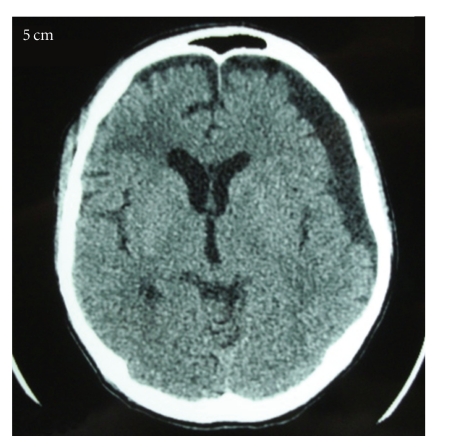
CT scan showing an increasing subdural fluid collection with mild mass effect and some effacement of lateral ventricle.

**Figure 2:**
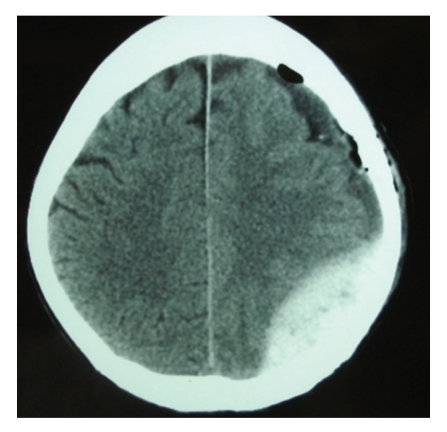
Postoperative CT scan demonstrating a large left parieto-occipital acute epidural hematoma.

A 38-year-old man suffered blunt head trauma in an automobile accident and was admitted to a local neurosurgical hospital. On admission, the patient was drowsy and his Glasgow Coma Scale score was 6, both pupils were isocoric and reactive to light. The patient underwent evaluation with computed tomography (CT) scan and basic laboratory studies. The patient had diffuse axonal injury grade III and was admitted in an intensive care unit(ICU). Two weeks later patient evolved with Glasgow Outcome Scale (GOS) 2. Three weeks after trauma patient had worsening level of consciousness, and right hemiparesis grade IV. A control CT scan was performed that showed an increasing subdural fluid collection with mild mass effect and some effacement of the left lateral ventricle (Figure 1). The fluid collection was most consistent with subdural hygroma unassociated with subdural hematoma. We perform a frontal trepanation with fast drainage of an extreme hypertensive subdural collection with citrine aspect. In postoperative patient return to ICU and in next day the patient remained in coma, underwent another CT scan that showed a large left parieto-occipital AEH (Figure 2). The patient was taken to the operating room. Craniotomy and evacuation of the hematoma were performed. Computed tomography after surgery revealed good surgical result. In hospital discharge patient presented GOS 3.

## 3. Discussion

SDG is a well-known complication of head trauma and neurosurgical procedures. Currently, treatment of SDG varies and can include observation, subdural tapping, subdural drainage, or placement of a subduropleural or subduroperitoneal shunt [3–7]. Indications for surgical management are somewhat nebulous but are generally based on the patient's overall clinical presentation. When SDG is associated with mass effect, as it was in the patient described in the present report, it should be considered a serious complication warranting neurosurgical intervention [7]. However, our case seems to be the first reported case with this postoperative complication. As a complication of surgery, AEH occurs mostly at craniotomy sites, with possible extension under the adjacent bone due to mechanical distension of the dura away from the skull. Extradural haematomas remote from the site of surgery, as in our case was more rare [8, 9].

The mechanism of remote postoperative AEH formation is unclear. We believe that in our case the etiology is similar to postoperative AEH of ventriculoperitoneal shunt. We believe that this patient, tapering long-standing elevated ICP by rapid release of subdural fluid will cause the brain collapse. This results in a suction force on structures between the cortex and inner table of the skull, that is, the arachnoid and dura mater [10]. Tearing of cortical bridging or meningeal vessels may lead to the formation of subdural or epidural hematomas, respectively. In case of the latter, detaching the collagenous fixation of the dura from the inner table of the skull may initially cause dural and diploic veins to bleed into the epidural space. As the hematoma enlarges and the distance between dura and bony arterial channels increases, dural arteries may also tear. Many authors thought that the traction exerted on the duramater by the many vessels attached to the brain causes a displacement and makes the vessels between the membrane and the skull to be torn [11, 12]. A sudden lowering of intracranial pressure, due to cortex collapse, helps hematomas increasing up to a catastrophic complication if not recognized and treated in time [11, 12]. 

 A high clinical suspicion or awareness of this entity is necessary to diagnose this dangerous disorder early. Complete reliance on neurologic monitoring, trust in an early CT scan, and a relative complacency after an apparently successful initial surgery for hygroma drainage may delay the diagnosis of this postoperative AEH and cause devastating consequences. We believe that cases like that of our patient indicate a clinically accurate monitoring, including simple procedures.
